# Editorial: Avoidance: From Basic Science to Psychopathology

**DOI:** 10.3389/fnbeh.2016.00015

**Published:** 2016-02-12

**Authors:** Richard J. Servatius

**Affiliations:** Neuroscience, Syracuse DVA Medical Center, Stress and Motivated Behavior Institute, Rutgers Biomedical Health SciencesNewark, NJ, USA

**Keywords:** avoidance learning, anxiety disorders, depressive disorder, research domain criteria, expectancy, animal models of mental disorders, association learning, escape

As a means of coping, avoidance encompasses thoughts and efforts toward prevention of future aversive experiences and events. Avoidance has been and remains controversial. Avoidance is accepted as a construct in many areas of research, but is roundly disdained in others. Why is such a critical feature of coping both acknowledged as such, but almost reluctantly studied?

For one, avoidance is often conflated with fear. Fear is an emotion. Threat conditions which engender fear also engender a host of physiological and behavioral responses (Ledoux, 2013). In animals, exposure to aversive stimuli or cues associated with aversive stimuli induce freezing, fleeing, or aggressive displays depending on the context of exposure—all behavioral manifestations of threat (Osada et al.). Responses to threat are relatively simple, engendered and refined through a circumscribed neural circuitry (Ledoux and Muller, 1997; Delgado et al., 2008). Fear and defensive responses to threat are readily and almost universally acquired. Those under threat (Shors and Servatius, 1997), stress (Servatius and Shors, 1994), and fearful (Mosig et al.) have a generalized facilitation of associative learning making threat and fear more pervasive. The engendering of fear and its expression is a highly researched concept; advancements in fear and the neurobiology subsuming fear is among the most notable and exhaustive neurobiological achievements in the past half century.

By comparison, avoidance is a fairly sophisticated construct. Avoidance is the situational evaluation of likelihoods, efficacy of responses, and costs. Avoidance is often weighed against alternatives; alternatives with differing or competing motivations (Beck et al., 2011; Fernando et al., 2014; Ilango et al.; Sheynin et al.). For many applications and circumstances fear and avoidance seem to be inseparable, so the terms become conflated. In the vernacular, fear is an immediate response to stressors and fear motivates avoidance. Therefore, in many circumstances those avoiding are *expected* to be experiencing fear. However, the empirical literature provides ample evidence that the processes are distinct (Bolles, 1968; Seligman and Johnston, 1973; Rio-Alamos et al.) and while the neurocircuitry, such as the lateral habenula (Shumake et al., 2010; Ilango et al., 2013) and cerebellum (Steinmetz et al., 1993) overlaps (Freeman et al., 1996, 1997; Bravo-Rivera et al., 2014; Campese et al.; McCue et al.; Jiao et al.), their influences on these processes potentially do not. Further distinguishing fear and threat from avoidance, septal (Thomas and Van Atta, 1972; Hedges et al., 1975) and hippocampal lesions (Cominski et al.) are known to *facilitate* avoidance acquisition, whereas these brain regions are critically involved in fear conditioning when intact (Kim et al., 1993; Desmedt et al., 1998; Knight et al., 2004).

As a research topic, avoidance all but disappeared through the 1990's, a phenomenon that has been noted in a number of recent reviews (Dymond and Roche, 2009; Krypotos et al.). Reduction in the study of avoidance stemmed from theoretical and practical considerations. In humans, the rise of institutional review boards and the reluctance of institutions and investigators to study reactions to aversive, painful stimulation or uncomfortable situations stymied progress. Added to these concerns, there were growing controversies regarding the role of awareness and instructional sets in human associative learning. Explicit information stemming from the consent form and instructions complicated experimental designs and interpretations of acquisition. Now there are but a few laboratories across the world with a vested interest in studying avoidance acquisition and extinction in humans, the TOPIC highlights several (Myers et al., 2013; Schlund et al., 2013; Sheynin et al., 2015; Cameron et al.; Moustafa et al.). Otherwise, avoidance and coping are primarily studied through self-report survey instruments which document coping strategies (Snell et al., 2011; Ayers et al., 2014).

In animals, the meteoric rise of electrophysiological and molecular techniques made reductionistic procedures ever more popular. This was in the face of Bolles formulation of species specific defense reactions (SSDRs) (Bolles, 1970). A reading of Bolles strongly suggests that the most popular applications of avoidance learning in animals were reducible to reflexes. Avoidance that relied on SSDRs would be difficult to distinguish from fear responses or their modification and would be better studied in clearer procedures. Bolles did not negate avoidance learning, but argued that avoidance was obscured by SSDRs and arbitrary avoidances provided clear evidence of avoidance, which would be slowly and incrementally acquired. The Bolles position muddled already difficult discussions concerning reinforcement in avoidance acquisition (Bersh, 2001; Dinsmoor, 2001; Hineline, 2001). The many criticisms of avoidance learning and its proper interpretation became more and more inaccessible to the average reader and more esoteric in argument. The zeitgeist is avoidance responses either a SSDR or require the suppression of SSDRs. SSDRs reflect fear and fear is more clearly examined in freezing (Fanselow and Poulos, 2005) or by examining its exaggeration of acoustic startle responses (Davis, 2006) under conditions in which control procedures are established to reveal associativity. Although arbitrary responses provide clear evidence of avoidance (Avcu et al.; Bravo-Rivera et al., 2014; Servatius et al.), these procedures became more and more unpopular. An increase in demand for throughput (self-contained, relatively short, and easily scored procedures) is at odds with the seemingly slow development of avoidance. In an unfortunate happenchance, “passive avoidance” remains in the parlance of behavioral neuroscience, but the high-throughput tasks and protocols to study “passive avoidance” are essentially assessing punishment.

Modern theorists of avoidance have moved away from response dynamics to cognitive processes driving response dynamics. Humans and mammals form expectancies. Avoidance expression reflects propositional knowledge but also the context in which knowledge is to be expressed (Seligman and Johnston, 1973; Lovibond et al., 2008, 2009; Dymond and Roche, 2009). Knowledge is subject to error and error correction (Myers et al.; Sheynin et al.). The difficulties encountered in learning arbitrary responses may not rest in how unnatural such responses are to humans and animals (Dinsmoor, 2001), but in the pressures of time/distance (Fanselow and Lester, 1988) and a cost/benefit analysis. There is a need for conceptual bridges between propositional knowledge central to expectancy models of avoidance and animal research in which processes are resolvable to response dynamics (response selectivity, strength of responding, and probability of responding; Krypotos et al.).

Recently, the National Institute of Mental Health (NIMH) in the United States embarked on research domain criteria (RDoC) to facilitate integration across levels of analysis and between diagnostic boundaries. The Negative Valence System encompasses acute responses to threat (fear) and inferred threat (anxiety), with escape/avoidance learning and expression emerging with sustained threat. In the NIMH working group discussion, ambivalence was expressed concerning whether sustained threat is distinct from acute threat, except for the time dimension. An undercurrent is that the sustained threat dimension, and by implication avoidance and escape, is not distinctive of acute conditions. The bounding conditions of avoidance are not only the duration of threat (acute/sustained), but the perceived intensity of threat, its perceived proximity, and the utility of responses or efforts. For perceived proximity of time, parametric manipulations of signal-shock intervals illustrate this point. Shuttling as the requisite response (a modified SSDR) is efficiently acquired with CS-US intervals of 10–20 s (Black, 1963). In lever press (not an SSDR) avoidance, escape behaviors predominate when signal-shock intervals are less than 20-s (Berger and Brush, 1975), with very few avoidance responses expressed after days of training (Servatius et al.). However, knowledge about avoidance is acquired; avoidance is not *expressed* (Servatius et al.). Using a crossover design those trained with a 10-s warning signal and exhibiting nominal avoidance rates displayed greater than 60% avoidance when switched to 60-s warning signal—nearly asymptotic performance of those trained initially with a 60-s warning signal. As to stressor intensity, shuttle box avoidance is efficiently acquired with foot shocks of moderately low intensity (0.2–0.5 mA) (Levine, 1966) with decrements apparent with shock intensity greater than 1.0 mA (Moyer and Korn, 1964). In contrast, lever press avoidance is efficiently acquired with shock intensities of 1.0–2.0 mA (Berger and Brush, 1975; Servatius et al., 2008; Avcu et al.). These features illustrate that avoidance acquired with arbitrary responses differ in a number of parameters from those modifying reflexive responses or “natural” responses, which are in turn distinct from fear responses. On the other hand, recent work also shows fear is more nuanced as fear contributes to sustained processes such as foraging (Kim et al., 2014).

In subsequent position papers concerning RDoCs, fear and threat processes feature prominently, whereas avoidance and coping do not (Cuthbert et al., 2003; Insel et al., 2010; Cuthbert, 2015). This is indeed unfortunate. An opportunity to intensify efforts in avoidance research is being missed. The mental health implications are extensive. Psychologically healthy coping strikes a balance between avoidance (responding in anticipation of aversive stimulation) and escape (responding in the presence of the stimulation) and competing motivations of approach (Ilango et al.; Ilango et al.). Deviant forms of avoidance are evident in autism (Richer, 1976), anxiety (Ly and Roelofs, 2009); (Kashdan et al., 2014), phobias, posttraumatic stress disorder (PTSD; North et al., 2004; Kashdan et al., 2009), major depression (Ottenbreit et al., 2014) and suicide (Dixon et al., 1991). Over-expression of avoidance, as in anxiety disorders and PTSD, insulates one from aversive thoughts or experiences at the expense of self-limiting interpersonal and environmental interactions. Under-expression of avoidance, as in depression or suicidality, unduly exposes one to aversive thoughts and experiences that would be otherwise controllable, severely depleting resources and progressing down a demoralizing spiral.

Diathesis models of mental illness capture avoidance biases as dynamic interactions of vulnerabilities (genes, epigenetics, personality, and developmental phases) with risk factors (psychological stressors, physical injuries) ultimately expressed as psychopathology. For example, behaviorally inhibited temperament, withdrawal in the face of social and nonsocial challenges, is a vulnerability factor for anxiety disorders (Moffitt et al., 2007). Humans expressing behavioral inhibition (BI) display enhanced avoidance expression (Sheynin et al.), and enhanced new motor learning (Caulfield et al.; Holloway et al., 2014), especially under degraded contingencies (Holloway et al., 2014; Allen et al.). Facilitated avoidance acquisition (Avcu et al.; Beck et al.; Jiao et al.; Servatius et al.) and new motor learning (Ricart et al., 2011a,b) are also apparent in Wistar-Kyoto rats, an animal model of BI temperament. Further, avoidance extinction is typically more difficult to obtain than extinction of fear. This is likely amplified by individual differences (Avcu et al.; Cominski et al.). Uncovering of neurobiological processes biasing avoidance expression and extinction has the promise of providing targets for individualized therapeutics and treatments for a number of psychopathological disorders.

Hence, there continues to be a need for an integration of human and animal research focused on coping and in particular avoidance coping. Model systems of avoidance that allow for bidirectional modifications of acquisition, expression, and extinction—protocols that allow for increased as well as decreased expression—are useful in translating basic science to psychopathology. By extension, RDoC constructs should be sensitive to individual differences, both accentuating and diminishing in the appearance of avoidance.

An open discussion of what features constitute fear, threat, anxiety, and avoidance would not only benefit basic science and psychopathology, but areas of research that are otherwise ignoring the infighting and are making substantial progress in improving health (e.g., fear-avoidance model of pain Vlaeyen et al., 1995; Crombez et al., 2012).

Approach Avoidance: Have no fear!

## Author contributions

The author confirms being the sole contributor of this work and approved it for publication.

## Funding

Supported by the Stress and Motivated Behavior Institute through funding from the Department of Defense, Armaments Research Development Engineering Center.

### Conflict of interest statement

The author declares that the research was conducted in the absence of any commercial or financial relationships that could be construed as a potential conflict of interest.
